# Supplementary material for: Time‑resolved multi-omic analysis of paclitaxel exposure in human iPSC‑derived sensory neurons unveils mechanisms of chemotherapy‑induced peripheral neuropathy
Source: Cell Death Dis. 2026 Feb 10;17(1):211. doi: 10.1038/s41419-026-08445-2 (PMC12921266; doi:10.1038/s41419-026-08445-2)
Supplement: Supplementary file 15 — Dataset 1. [file 41419_2026_8445_MOESM15_ESM.zip › CDD_Open_Data/04_RNA_Seq/Timeline_panel_plots_etc_2023-01-23_1609.zip!/Panel_Plots_etc/Evidence_Plots/6h/evidence_plot_standard contrasts_tmod_6h_PTX_vs_DMSO_ID2_DC.M5.9_Protein Synthesis.pdf]

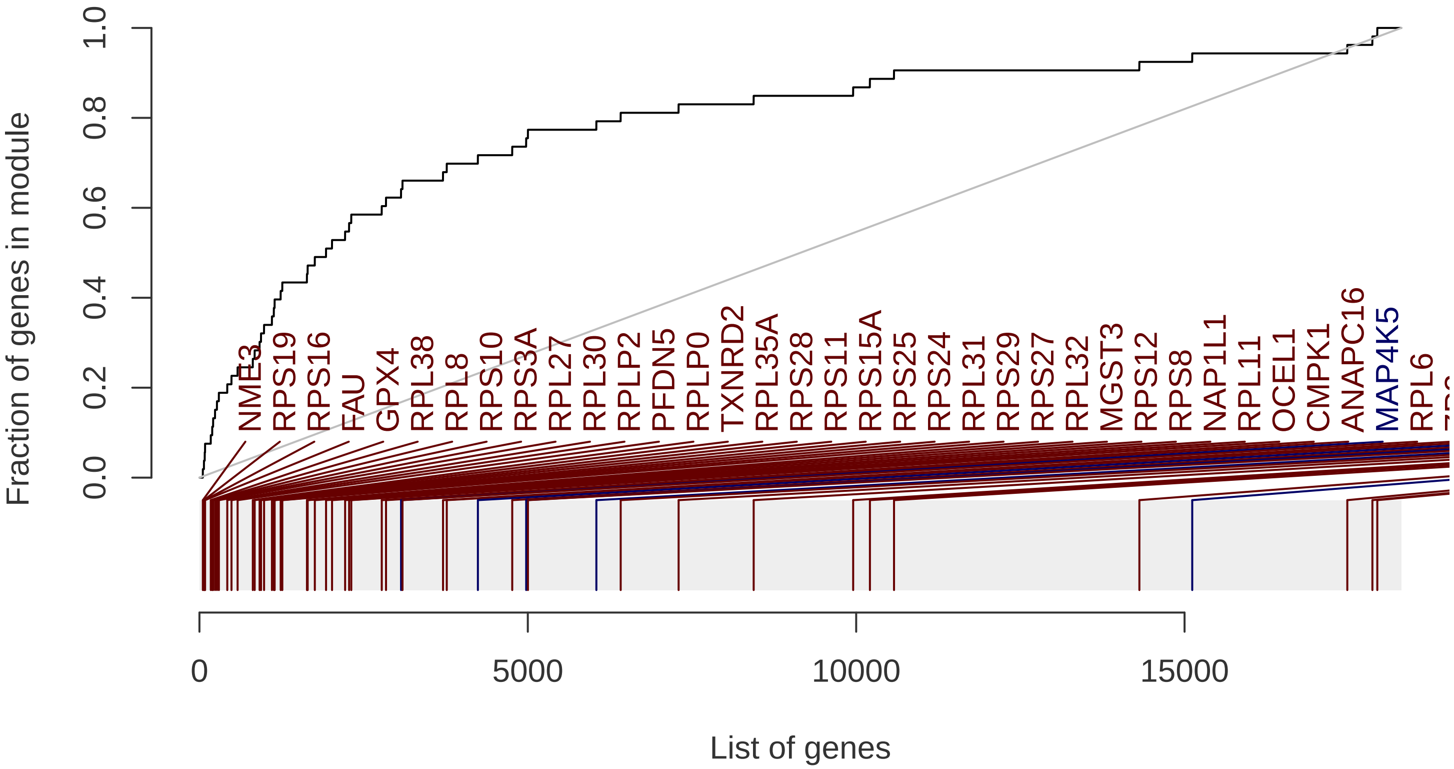

Fraction of genes in module
0.0
0.2
0.4
0.6
0.8
1.0
NME3
RPS19
RPS16
FAU
GPX4
RPL38
RPL8
RPS10
RPS3A
RPL27
RPL30
RPLP2
PFDN5
RPLP0
TXNRD2
RPL35A
RPS28
RPS11
RPS15A
RPS25
RPS24
RPL31
RPS29
RPS27
RPL32
MGST3
RPS12
RPS8
NAP1L1
RPL11
OCEL1
CMPK1
ANAPC16
MAP4K5
RPL6
0
5000
10000
15000
List of genes
